# Facial Masculinity: How the Choice of Measurement Method Enables to Detect Its Influence on Behaviour

**DOI:** 10.1371/journal.pone.0112157

**Published:** 2014-11-12

**Authors:** Santiago Sanchez-Pages, Claudia Rodriguez-Ruiz, Enrique Turiegano

**Affiliations:** 1 Departament de Teoria Econòmica, Universitat de Barcelona, Barcelona, Spain, and Edinburgh School of Economics, Edinburgh, United Kingdom; 2 Departamento de Biología, Universidad Autónoma de Madrid, Madrid, Spain; Utrecht University, Netherlands

## Abstract

Recent research has explored the relationship between facial masculinity, human male behaviour and males' perceived features (i.e. attractiveness). The methods of measurement of facial masculinity employed in the literature are quite diverse. In the present paper, we use several methods of measuring facial masculinity to study the effect of this feature on risk attitudes and trustworthiness. We employ two strategic interactions to measure these two traits, a first-price auction and a trust game. We find that facial width-to-height ratio is the best predictor of trustworthiness, and that measures of masculinity which use Geometric Morphometrics are the best suited to link masculinity and bidding behaviour. However, we observe that the link between masculinity and bidding in the first-price auction might be driven by competitiveness and not by risk aversion only. Finally, we test the relationship between facial measures of masculinity and perceived masculinity. As a conclusion, we suggest that researchers in the field should measure masculinity using one of these methods in order to obtain comparable results. We also encourage researchers to revise the existing literature on this topic following these measurement methods.

## Introduction

Recently, a significant number of scientific and non-scientific articles have highlighted the poor record of successful replications of scientific results [1]–[6], especially in behavioural sciences [7], [8]. There are multiple and complex reasons behind this problem, from statistical mistakes to publication bias. A very relevant, and easily solvable, issue is the use of stereotyped analytical methods [9]. Adherence to common standards, protocols and measurement methods are likely to increase the proportion of true, and thus replicable, findings. To the very least, the standardisation of practices can contribute to generate comparable results.

In the present work, we aim to contribute to this goal within the study of masculinity and its influence on behaviour. Masculinity, defined as the quality of having masculine physical traits, has become an important element in the research on male human behaviour. This variable has been shown to correlate with several behaviours and characteristics (as in [10]–[12]), especially in relation to attractiveness (reviewed in [13]–[15], but see [16]–[18]). But although masculinity has become an important variable in many different fields of behavioural sciences, the number of methods used to measure it is roughly similar to the number of research teams working in the field.

Masculinity is likely to be related to males' exposure to testosterone (T) during puberty. Exposure to T during development produces several changes in the male body, such as a greater musculoskeletal development and the rise of secondary sexual characteristics. It also affects males' nervous system [19]. Thus, exposure to T influences both human male behaviour [20] and their physical appearance [21], [22]. Hence, one should expect a correlation between the level of physical masculinization and the degree of “behavioural masculinity” as both are affected by exposure to T during development.

Variables related to high T exposure, from current T levels to low second to fourth digit ratio (2D:4D), are positively linked to bolder behaviours in men. Circulating T has been described as linked to status-seeking behaviours, aggressiveness, sex drive, and risk-taking [22]–[27]. 2D:4D is linked to T levels during phoetal development [28] and it is related to aggressiveness and competitiveness [29]–[31]. Masculinity, and facial masculinity in particular, is also related to other features and behaviours, such as perceived trustworthiness [32], aggressiveness and dominance [33], [34], risk-taking [10], the tendency to cooperate and to self-sacrifice when competing against out-groups [12], and deception [11].

The link between exposure to T and these behaviours is usually attributed to one of the reproductive functions of this hormone in males, directing male behaviour towards increasing reproductive success. Related to this role, exposure to T has been considered a good predictor of male attractiveness (reviewed in [13]–[15]), as conjectured by the immunocompetence hypothesis [15], [35], [36]. However, the relationship between masculinity and attractiveness is still under scrutiny, since some authors find a positive correlation between them [37], [38], whereas others do not [17], [18], [39]. These mixed results might be due to the interference of other variables such as aggressiveness [16].

There exists a wide variety of measures of masculinity at the disposal of researchers in this field. One of the most prominent measures is perceived masculinity as rated by a sample of males and/or females [17], [40]–[43]. However, perceived masculinity is not always a good measure of T levels during development. Exposure to T during puberty has, indeed, an impact on the facial shape. But perceived masculinity is also influenced by features such as perceived health or skin color [39]. Because of this, alternative measures are needed if the researcher wants to isolate the effect of the level of exposure to T during adolescence on adult behaviour.

The calculation of morphometric measures of facial masculinity can employ sample-independent or sample-dependent techniques. Among the sample-independent ones, some methods take simple measurements from males' faces [11],[12],[33],[34],[44]–[46], whereas others use indexes constructed from these measurements [10], [18], [47]-[50]. Sample-dependent methods [39], [51]–[54] are based on statistical techniques performed on a sample of subjects. These methods generate measures which depend on the female reference sample employed. Some morphometric techniques produce measures of masculinity which correlate with perceived attractiveness [32], [44], [47], [48], [52], but others do not [15], [18], [39]. Similarly, some measures correlate with perceived features like male facial trustworthiness [32], dominance [33] or masculinity [52], but others do not correlate with either perceived masculinity or dominance [50]. One of the most widely employed measures of masculinity is facial width-to-height ratio (fWHR) [32], [34], [46], [55]–[59], which correlates with masculine behaviours such as aggressiveness and dominance [34]. However, it remains unclear whether fWHR is a sexually dimorphic trait [55]–[58], since the dimorphism previously observed could be due to the interference of other traits such as the body mass index (BMI) [45].

In the present work, we study the relationship between a set of measures of masculinity employed by different authors and two features previously described as linked to masculinity: risk attitude and facial trustworthiness. To this aim we explore the link between these measures of masculinity and the behaviour displayed in two experimental settings: a first-price auction with private values and a simplified version of the trust game. In addition, we explore the relationship between these measures and perceived masculinity as rated by an external group.

A first-price auction is a version of the Dutch or descending price auction in which participants bid to obtain an object and the highest bidder wins and pays a price equal to his posted bid. This auction is with private values, that is, participants' valuations of the auctioned object are independent of each other and unknown to other participants. In a first-price auction with private values there exists a fundamental trade-off: by increasing his bid, a participant is reducing the risk of losing the object but he pays a higher price in case of winning. Therefore, behaviour in a first-price auction should theoretically depend on risk attitudes [60], [61]: More risk-averse bidders should post higher bids in order to avoid the risk of losing the object [62]. It has been shown that there is a negative correlation between risk aversion and current T levels [24], [63], [64], although the association seems to be nonlinear [65]. Risk aversion is, in turn, negatively correlated with traits such as the 2D:4D ratio [66] and facial masculinity. Experimental evidence shows that males with high facial masculinity are more prone to take risks when investing money [10]. The relationship between masculinity and risk aversion could be mediated by the rearrangements on neural circuits caused by exposure to T during adolescence [19] or by the current T level itself [41], [42], [50]. Given the relationship between facial masculinity, T and risk aversion, measures of facial masculinity should display a negative correlation with bids in a first-price auction: more masculine males, being less risk averse, should post lower bids. We check whether the different measures of facial masculinity we consider display this conjectured relation between masculinity and bidding behaviour.

Some authors have also linked behaviour in auctions to competitiveness, understood as the “desire to win” rather than as a “competitive motivation” (these concepts are commonly confused in the literature [67]). This influence of competitiveness on bidding behavior seems to be behind the so-called “winner's curse” [68]–[70] described in common value auctions and behind the “auction fever”, described in ascending auctions [71]. However, it must be pointed out that there exist some fundamental differences between those auctions and the one used in this work. In common value auctions, the value of the object is the same to all participants, who can only base their bids on their own estimate of this value. On the other hand, in live and internet ascending auctions [71] participants could bid more than once. Social context is more relevant and thus more likely to promote competitiveness in these two types of auctions [68], than in our first-price auction where social considerations were intended to be minimal.

Although with several variations [32], [72], the basic trust game entails two participants, an investor and a trustee. The investor must decide whether to transfer an amount of money to the trustee. If this is the case, that trustee receives that amount multiplied by a factor greater than one (typically three or four). Then, the trustee has to decide whether to keep that increased amount of money or to return part of it to the investor. Usually, investors are matched with a number of trustees and are presented their photograph. Then, investors can base their decision whether to trust or not the trustee on his/her face. Previous results show that men with more masculine faces appear as less trustworthy to others [32], and that they are indeed more likely to exploit the trust of others [11]. Thus, measures of facial masculinity should in principle correlate negatively with the likelihood of being trusted by others, i.e. trustworthiness.

Finally, we also compare the different measures of facial masculinity with perceived masculinity. This comparison is a relevant exercise given that the link between measured and perceived masculinity is currently under scrutiny [15], [37]–[39], [50], [52].

## Methods

Our subject pool was composed by 147 self-declared white male students from Madrid (n = 78) and Edinburgh (n = 69). They were aged from 17 to 30. The Madrid students (Mean ±SEM; 21.04±0.28 yr) were significantly older (*t* test: t_145_ = 4.534, p<0.001) than the Edinburgh ones (19.52±0.17 yr). This subject pool constitutes the sample we used in a previous study [54].

### Ethics Statement

The experimental protocol, including collection of photographs, was approved (reference number: CEI-27-642) by the relevant ethics committees at University of Edinburgh (Business School Research Ethics Committee) and at Universidad Autónoma de Madrid (Comité de Ética de la Investigación). Written consent was obtained from all participants and from the parents of the two underage participants.

### Photos

Three full frontal facial color photographs were taken of all participants with an Olympus E-500 digital camera with resolution 3264×2448 in JPEG format. The photos were taken under strictly standardised conditions of illumination, camera distance and zoom. Participants had to remove any facial adornment and maintained a neutral expression looking directly into the camera. From the three images of each participant we chose the best one for our purposes.

### Measures of facial masculinity

We computed participants' facial masculinity following a variety of methods previously employed in the literature. Below, we briefly describe these measures and the methods used to construct them.

Some measures of masculinity focus on a single facial feature that has shown significant gender differences. One of the most frequently employed among these is the width to upper face height ratio or fWHR [32], [34], [59]. Width is calculated as the maximum horizontal distance from the left to the right zygion (bizygomatic width) of the facial image. The upper face height is calculated as the vertical distance between the lip and brow of the same facial image. Some authors have found a correlation between fWHR and male facial trustworthiness [32] and male aggressiveness [34]. Others do not find this correlation [58]. fWHR has traditionally been thought to be sexually dimorphic in humans [59]. However, many authors have failed to find significant differences between males and females, both using Carré and McCormick's (2008) method [34] with a sample of 470 individuals [55], or similar methods of calculation with even larger samples [56]–[58].

Another measure of facial masculinity based in a single trait is the eye-mouth-eye (EME) angle. Some authors argue that EME is a sexually dimorphic trait, significantly smaller in males [44], although this sexual dimorphism has also been questioned [73]. This measure only shows a slight correlation with interpupillary distance and upper face height even though both features define this angle.

Some authors have considered simultaneously several of these sexually dimorphic features. For example, Burriss and collaborators [33] measured a number of facial features in order to test their correlation with 2D:4D. They employed three measures that were significantly different between sexes: upper lip height (ULh, lower in men), lower lip height (LLh, lower in men) and nose width (Nw, larger in men). All their measures were rendered as a percentage of interpupillary distance.

Other methods of measuring masculinity integrate several measures of sexually dimorphic features in an index. One index [47] simply adds up standardised measures of cheek-bone prominence and lower face length (Index 1). Another index frequently employed [48], [50] adds five facial measures that show dimorphic differences between sexes (eye length, lower face height/face height, cheekbone prominence, face width/lower face height and mean eyebrow height, all of them divided by interpupillary distance). This index (Index 2) yields higher scores when these features are more masculine (smaller eyes, smaller eyebrow distance, smaller cheekbone prominence, smaller face width and larger lower face). A modified version of this index includes jaw height/lower face height and excludes eyebrow height and eye [10], [49]. This index (Index 3) combines linearly these four measures after standardisation (i.e., [JH/LFH + LFH/FH]−[ChP + FW/LFH]) in order to obtain a measure of masculinity.

A third possibility is to measure masculinity by comparing a sample of male subjects with a sample of females in order to obtain a measure which differentiates masculine from feminine faces. We are aware of three measures of this kind. Two of them employ Geometric Morphometrics techniques. The advantage of Geometric Morphometrics measurements is that they incorporate the complete geometric information contained in the facial shape [74]–[77]. They are based on a number of landmark coordinates placed directly on the face rather than on distances or angles (usually calculated from some of these landmarks). Geometric Morphometrics avoids some of the well documented problems of “traditional” Morphometrics [74], [77], [78].

**Figure 1:**
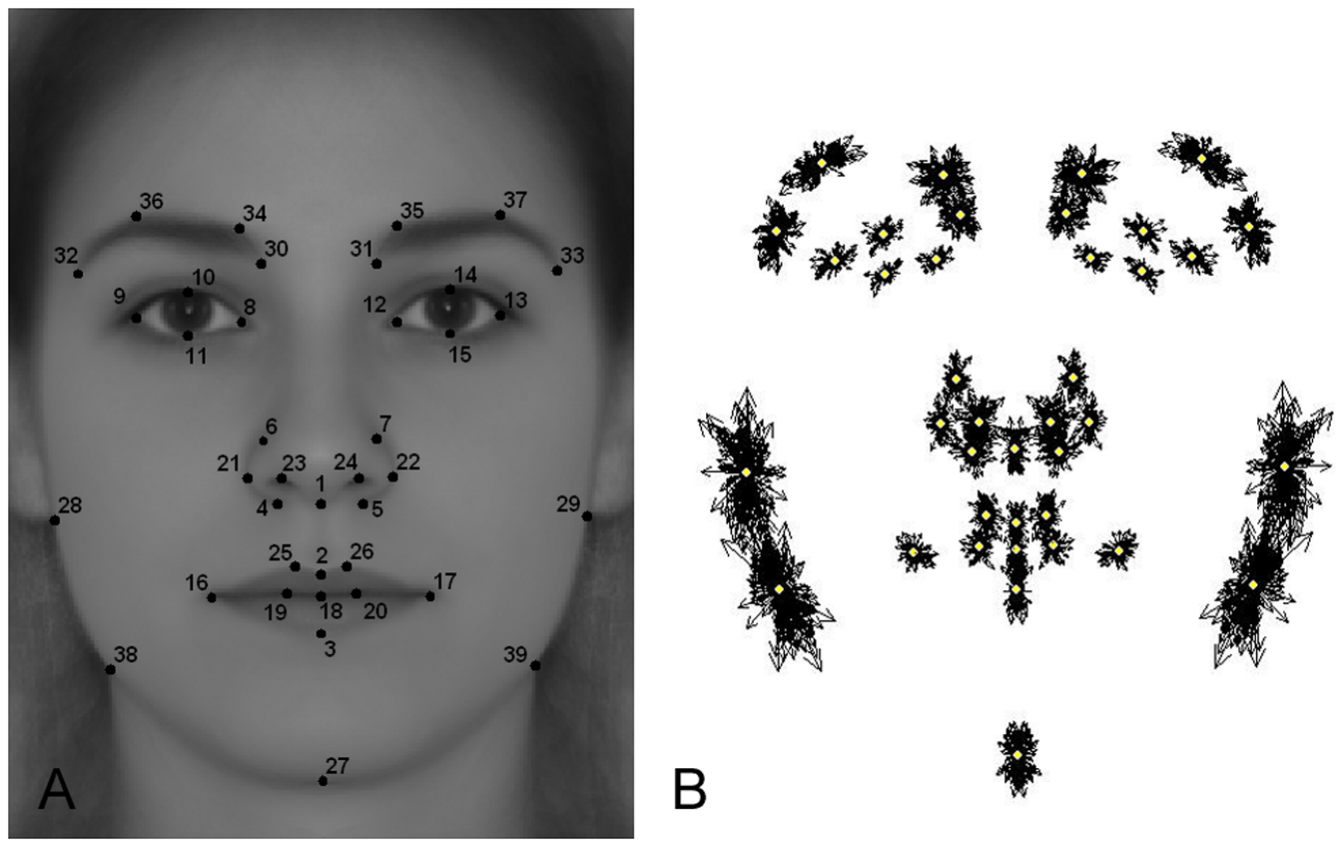
Employed landmarks. A) An average face generated with the complete female population (n = 74) and the 39 landmarks placed. B) All 147 subjects' landmarks configurations superimposed after Procrustes Fit.

One possibility to measure facial masculinity employing Geometric Morphometrics is to calculate the Procrustes distance between the shape of the symmetrized participant's faces (males) and a reference feminine face (as in [54], [79]). The lower this Procrustes distance is, the closer the participant's face is to the reference female face. To compute this measure, the shape of each face has to be defined by manually setting predetermined points called landmarks (LMs). These LMs have to be unambiguously identified in every photo (See Figure 1) and must be placed in positions that ensure a reasonable degree of correspondence between LMs locations across images [75]. Given that we are interested in changes on facial shape caused by T during adolescence, LMs are not placed on soft parts of the face, which are more prone to variations during life [80]–[83]. Symmetrized photos of males (average of mirror images) are employed in order to avoid the inclusion of any indirect measure of symmetry, given that the female reference face is completely symmetrical. We built the female reference image by averaging photos of 74 female students of the same participants' age (20.36±0.15 yr) and location (48 from Madrid and 26 from Edinburgh). Procrustes distance (ProcDist) was calculated using the TPS software package (by F.J. Rohlf; see http://life.bio.sunysb.edu/morph/). This measure of masculinity is not correlated with facial fluctuating asymmetry (Pearson correlation coefficient: r_147_ = 0.059; p = 0.475). We also tested whether the configuration defined by the 39 LMs discriminates accurately between symmetrized males and females in the sample. Discriminant function scores could correctly classify the sex of 95.48% of the faces (T^2^ test: T^2^ = 1052.1578; p<0.0001).

Another possibility is to employ Geometric Morphometrics to obtain a discriminant function that can be used as a measure of masculinity (as in [39]). The LMs placed to compute the previous measurement are also used to compute this one. MorphoJ software (see http://www.flywings.org.uk/MorphoJ_page.htm) superimposes the shapes with a generalized least-squares procrustes fit. The covariance matrix across individuals is then computed from these data, and a PCA is carried out on it. For subsequent analyses, we choose the first eight PCs, which altogether account for 83.25% of the variance in facial landmark configuration. Step-wise discriminant analysis is then used to choose among those PCs which better discriminate between sexes. The resulting discriminant function incorporates three of the PCs and classifies correctly 90.50% of the faces. The discriminant function scores constitute an index of masculinity (DiscSco1), with smaller scores corresponding to more masculine faces.

Finally, a third method to compare male faces to a female face of reference is to perform a PCA from several facial measures (different between sexes) and include the significant factors in a discriminant analysis. These discriminant scores are employed as a measure of masculinity [51]–[53]. We followed the procedure described in [51]. We took the same ten different facial measures (Face length, Face width, Chin length, Eye height, Eye width, Interpupillary distance, Lip height, Lip width, Jaw width and Face length minus chin). Then we derived scores to control for face size by computing the non-standardised residuals from regressions on the proper face measurement (see [51] for more details). We examined sex differences by GLM (controlling for age) of the eight residual variables (147 males and 74 females). Like Gangestad and Thornhill [51], we found that five of these measures significantly discriminate between sexes (chin length, jaw width, eyes length and width and lips width). In addition, we found significant differences for face width. We then performed a principal axis factor analysis on these six variables. In our case, there were three major factors which accounted for 73.00% of the variation (the first five eigenvalues  = 1.814, 1.466, 1.100, 0.845 and 0.453). We rotated (varimax) and extracted the factors. The first component was primarily defined by eye width (pattern matrix loading  = 0.815), lip width (0.643) and face width (negatively; −0.579). The second factor was defined mainly by jaw width (0.895) and chin length (0.808). Finally the third factor contributors were mainly eye height (0.847) and face width (0.713). These three factors significantly discriminated between sexes (respectively, PC1: F_1,219_ = 3.726, p = 0.055; PC2: F_1,219_ = 5.245, p = 0.023; PC3: F_1,219_ = 10.058, p = 0.002). We input the three factors in a discriminant analysis predicting sex. Discriminant function scores correctly classified the sex of 70.6% of the faces (these scores correlated 0.446 with the first factor, 0.528 with the second and 0.723 with third). Discriminant function scores, with low values corresponding to males, were used as a measure of facial masculinity (DiscSco2).

In addition, we analysed the robustness of the masculinity measures which are sample-dependent (ProcDist, DiscSco1 and DiscSco2). We find that these measures of masculinity are relatively independent of the sample employed to build the female reference face (see Appendix S1). We also tested for changes in the set of LMs chosen to compute measures which employ Geometric Morphometrics. We observed that a small change in the number of LMs does not seem to affect these measures too much, although removing a few specific LMs has a substantial impact on its link on the studied behaviours (see Appendix S1).

We tested for gender differences in all these masculinity measures. With the exception of Lower Lip Height (LLh), all of them are sexually dimorphic and follow the differences described in literature (see Table 1). All morphometric variables follow a normal distribution except ULh. We log-transformed ULh in order to fit the assumption of normality. This allowed us to perform parametric tests with all these variables. Correlations between all the masculinity measures employed are presented as Appendix S1.

**Table 1:** Summary statistics.

	Females	Males	
**fWHR**	2.047±0.015	2.090±0.013	t_219_ = −2.080 p = 0.039
**EME**	48.888±0.261	48.031±0.261	t_219_ = 2.080 p = 0.039
**ULh**	10.258±0.222	9.520±0.180	t_219_ = 2.472 p = 0.014
**LLh**	15.378±0.299	15.284±0.270	t_219_ = 0.217 p = 0.828
**Nw**	54.617±0.389	57.901±0.340	t_219_ = −5.931 p<0.001
**Index 1**	−0.285±0.139	0.144±0.116	t_219_ = −2.251 p = 0.025
**Index 2**	−1.216±0.252	0.612±0.204	t_219_ = −5.400 p<0.001
**Index 3**	−1.245±0.244	0.627±0.610	t_219_ = −5.454 p<0.001
**ProcDist**	6.339±0.212⋅10^−2^	8.467±0.177⋅10^−2^	t_219_ = −7.317 p<0.001
**DiscSco1**	1.787±0.112	−0.899±0.084	t_219_ = 18.842 p<0.001
**DiscSco2**	0.425±0.133	−0.214±0.076	t_219_ = 4.482 p<0.001

Values are presented as Mean ±SEM. Abbreviations: fWHR [34]; EME [44]; ULh, LLh and Nw [33]; Index 1 [47], Index 2 [48], [50], Index 3 [10], [49], ProcDist [54], [79], DiscSco1 [39], DiscSco2 [51].

### Perceived masculinity

The masculinity of our 147 subjects was rated by 36 older males (31.17±0.66 yr). We chose older males in order to prevent any kind of competition when rating. We did not use female raters in order to avoid the variability possibly caused by the phase of their menstrual cycle [84]. Subjects' photos were divided randomly into seven pools with 21 photos each. These pools were presented to raters in different days. We asked them to rate the masculinity of the participants' photos in a 1–7 scale (being 7 the most masculine and 1 the least masculine). The 36 raters displayed high internal consistency and reliability (Cronbach's alpha  = 0.98). The average of the individual scales was used as the measure of perceived masculinity. This variable is normally distributed, allowing us to perform parametric tests. The perceived masculinity was 3.98±0.11. There were no differences in the perceived masculinity scores between the populations of Madrid and Edinburgh (t_145_ = 0.428; p = 0.669).

### Bidding behaviour and risk aversion measure

Participants were asked to take part in a first-price auction with private values. Subjects had to bid for an object they were told they would be able to resell for 80 points (the exchange rate was 1 point equal to 1penny/1cent). Subjects were also told that they were in competition for the object with another person but they did not know how much the other person valued it (i.e., the amount the other person would be able to resell the object for). They were just told that the other person's valuation could be any amount of points between 0 and 100 with equal probability (technically, the valuation was uniformly distributed over the integers in the interval [0,100]). Subjects were told that the person with the highest bid would win the object. Hence, the higher the bid the more likely subjects were to acquire the item. Lower bids increase the net benefit from obtaining the object (the difference between the resell price of 80 and the bid) but they increase the risk of losing it (as it is more likely that the other participant will post a higher bid). The standard solution concept for this class of games is the Bayes-Nash equilibrium [62]. In this case, this theoretical prediction yields that risk-neutral participants should bid 40 (half their valuation) and that increasingly risk-averse participants should post bids increasingly close to 80. Hence higher bids are theoretically associated with higher levels of risk-aversion.

In order to test whether there is a link between risk aversion and bidding behaviour, we also measured risk aversion with the widely employed method proposed by Holt and Laury [85]. Subjects were asked to choose between two different monetary lotteries. The first lottery (Option A) entailed prizes of 80 and 100 points. The second lottery (Option B) entailed prizes of 5 and 200 points. Hence Option A was less variable. There were eleven of these choices which were increasing in the probability attached to the highest outcome within each option (and thus equally decreasing on the probabilities attached to the lowest outcomes). Hence, more risk-averse subjects should select a higher number of consecutive Option A choices, with risk-neutral subjects picking Option A in the first four choices, and risk-loving participants switching to Option B earlier on. This measure of risk aversion is widely employed in the literature, but its validity is currently under scrutiny [86].

The experiments were performed employing the z-Tree 3.2.10 software for economic experiments [87]. They were run in sessions with less than 20 subjects each. Before each session, subjects were carefully instructed about the experiment and their photographs were taken. All the subjects filled a questionnaire asking their age, sexual orientation, ethnicity and degree. They also received an official receipt they had to fill and return to the experimenters in order to receive their payment. At the beginning of the session, subjects were told that they were going to be paid according to some of the decisions they were going to take during the session. In order to avoid interference with the results, subjects were told about the exact method of payment computation when the session concluded. The experimental sessions took less than an hour.

The average bid made was of 59.97±1.17, ranging from 10 p to 85 p (median  = 60). This is consistent with an extensive body of experimental evidence showing that bidding behaviour in first-price auctions is consistent with significant levels of risk aversion [60]. Bids did not follow a normal distribution. There were no significant differences in the bids made between the populations of Madrid and Edinburgh (t_145_ = −0.232, p = 0.817). We asked subjects not to answer the risk-aversion test if they thought that they did not fully understand the meaning of the lotteries. Six of our subjects did not answer this test. Risk aversion scores classify 30.50% of subjects as risk-neutral and 57.45% as risk-averse. We found a weakly statistically significant positive correlation between the bid and Holt and Laury's measure of risk aversion (r_141_ = 0.151; p = 0.074).

### Male facial trustworthiness

To measure trustworthiness we employed a simplified version of the trust game [88]. In this version of the game, a participant called “the investor” is endowed with 50 points (the exchange rent was 1 point/1 eurocent). The investor then has to decide whether to transfer 30 of these 50 points to another participant, called “the trustee”. If the investor decides to transfer the points, the transferred points quadruplicate and the trustee receives 120 points. At that point, the trustee has to decide whether to keep these 120 points (so the investor is left with 20 points) or to return half of them to the investor (so the investor obtains 80 points). In the standard game theoretical prediction for this game, trustees do not return any money and consequently investors decide to keep the initial amount for themselves. However, experimental results show a substantial departure from this prediction; investors often trust trustees and trustees frequently reciprocate [88].

In our experiment, 21 participants (none of which had played as trustees) aged between 20 and 34, (27.48±1.17 yr) were asked to take part as investors. They had to decide individually whether to transfer their points or not to each of the 147 males whose photos were presented to them. 40 of these 147 males had actually played the game as trustees in a previous experiment. The other 107 participants had not played the game. The 40 participants who had played as trustees in the earlier study were asked whether they would return half of the 120 points to an anonymous male investor or whether they would keep the whole sum. The 21 participants acting as investors knew that their payoff from the game would be computed based on the combination of their own choice and the choice as trustees of some of the males whose photographs were presented to them, but they did not know which of the 147 subjects had actually played as trustees. The session was carried out in four series, with a pause between each series in order to allow participants to maintain their concentration. They were told about the exact method of computing payments when each session concluded. These experimental sessions took about an hour.

The 21 investors were consistent in their decision to trust (Cronbach's alpha  = 0.74). Trustworthiness scores for each of the 147 participants were calculated as the proportion of the 21 investors who considered them trustworthy trustees, i.e. transferred points to them. The average trustworthiness score was 0.476±0.016, ranging from 0.048 to 0.905. These scores were not normally distributed. There were no significant differences between trustworthiness scores obtained for the populations from Madrid and Edinburgh (t_145_ = −0.261, p = 0.795).

### Statistical analysis

Most tests (Student t test, Pearson correlation coefficient, Spearman rho correlation coefficient, principal axis factor analysis, discriminant analysis) were calculated employing SPSS15. Comparisons between correlation coefficients were performed as described in [89]. Morphometric analyses were performed using Morpho-J software. This software can run several multivariate test to compare shapes, providing significance levels by employing both parametric and permutation tests. We chose the last one in order to avoid problems with the assumptions of multivariate normality and equal covariance matrices, given that they are difficult to assess with morphometric data (because of high number of variables and small sample sizes). In order to compare the facial shape of males and females, the program run the T^2^ test, a multivariate equivalent of the univariate t test. The program ran 10000 rounds of random reallocations of the observations. In the discriminant analyses, the percentages of correct categorisation of photos were taken from cross validation classification.

## Results

Table 1 shows descriptive statistics for the different measures of masculinity. All measures, indexes and scores show significant differences between males and females except LLh (t_219_ = 0,217; p = 0,828). Table 2 shows correlations between the different measures of masculinity and perceived masculinity, the bid and the trustworthiness score. Age has also been included in the analysis.

**Table 2:** Correlations between perceived masculinity, bid and trustworthiness scores with morphometric measures (n = 147).

	Perceived Masculinity	Bid	Trustworthiness
**Age**	**r = 0.395**	ρ = −0.152	ρ = −0.009
	**p<0.001**	p = 0.067	p = 0.911
**fWHR**	r = 0.081	ρ = −0.082	**ρ = −0.339**
	p = 0.330	p = 0.322	**p<0.001**
**EME**	r = 0.064	ρ = −0.068	**ρ = −0.274**
	p = 0.444	p = 0.411	**p<0.001**
**Ln ULh**	r = −0.093	ρ = 0.091	ρ = −0.037
	p = 0.264	p = 0.272	p = 0.657
**LLh**	**r = −0.334**	ρ = 0.025	ρ = −0.154
	**p<0.001**	p = 0.760	p = 0.063
**Nw**	r = 0.033	ρ = −0.045	**ρ = 0.239**
	p = 0.691	p = 0.589	**p = 0.004**
**Index 1**	**r = 0.202**	ρ = −0.067	ρ = −0.058
	**p = 0.014**	p = 0.418	p = 0.487
**Index 2**	**r = 0.250**	ρ = −0.005	ρ = 0.114
	**p = 0.002**	p = 0.951	p = 0.168
**Index 3**	**r = 0.291**	ρ = 0.044	**ρ = 0.194**
	**p<0.001**	p = 0.601	**p = 0.018**
**ProcDist**	r = 0.111	**ρ = −0.164**	ρ = 0.074
	p = 0.180	**p = 0.047**	p = 0.374
**DiscSco1**	**r = −0.303**	**ρ = 0.182**	ρ = 0.040
	**p<0.001**	**p = 0.027**	p = 0.634
**DiscSco2**	**r = −0.390**	ρ = 0.044	ρ = 0.037
	**p<0.001**	p = 0.601	p = 0.658
**Perceived Masculinity**		ρ = −0.153	ρ = 0.074
		p = 0.064	p = 0.374

First of all, let us focus on the correlations between perceived masculinity and the masculinity measures (Table 2). As expected, Index 1, Index 2 and Index 3 correlate positively while DiscSco1, DiscSco2 and LLh correlate negatively. These six correlation coefficients are not significantly different among them (χ^2^_5_ = 3.713; p = 0.591). Age also shows a positive correlation with perceived masculinity.

Because bids and trustworthiness scores were not normally distributed, we calculated the Rho Spearman coefficients to analyse their correlation with the different masculinity measures (Table 2). Bids show a negative correlation with ProcDist and a positive one with DiscSco1. Thus we can conclude that risky bidding behaviour correlates positively with masculinity, as males show larger values in ProcDist than females, but show lower values for DiscSco1 (Table 1). The two correlation coefficients are not significantly different (Z = 0.157; p = 0.875). After correcting for multiple testing, these correlations became non-significant. Note however that the aim of the present work is not to uncover new relationships but to establish which measures of facial masculinity are robustly associated with behavior.

Given that none of these masculinity measures yields a significant correlation with Holt and Laury's measure of risk aversion (see Appendix S1), we explored whether variables other than risk aversion could explain the observed correlations with bidding behaviour. To this aim, we estimated two linear regression models with bidding behavior as dependent variable and including these two variables (ProcDist and DiscSco1) and controlling for risk aversion. Results show that both morphometric measures of masculinity have a strong effect on bidding behaviour (ProcDist:β = −0.252, p  = 0.002; DiscSco1: β = 0.239, p = 0.004). Residuals for both regressions are normally distributed.

On the other hand, trustworthiness scores correlate negatively with fWHR and EME, and positively with Nw and Index 3. Hence, trustworthiness is negatively associated with masculinity as measured by fWHR, but it correlates positively with masculinity as measured by EME, Nw and Index 3. The correlation coefficients of trustworthiness with these three measures are not significantly different (χ^2^_2_ = 0.519; p = 0.772).

## Discussion

The main objective of this work was to analyse different methods of measuring facial masculinity in order to standardise the methodology employed to compute this feature. To this aim we have employed several methods of measuring facial masculinity. We studied how these different measures are related to bidding behaviour in a first-price auction, to trustworthiness in a trust game, and to perceived masculinity. As facial masculinity has been previously linked to all these three variables, we expected at least some of the masculinity measurements to correlate with them. Our main interest is to clarify which of the different measurement methods are more suitable to analyse the association between masculinity and different behaviours.

We chose to employ bidding behaviour in a first price auction as a measure of risk attitude for two reasons. First, because it is theoretically related to risk taking [60]–[62] and it has been previously employed in this sense [90]–[92]. Second, because T usually promotes behaviours aimed to increase or maintain individual status [93]. We thus expected that the effect of T on behaviour would become more salient in strategic interactions such as the first price auction where a prize is clearly at stake between two individuals. As we postulated, some measures of facial masculinity show a significant correlation with bidding behaviour. Specifically, bids made show a negative correlation with masculinity as measured by employing Geometric Morphometrics (ProcDist and DiscSco1). We postulated this relationship only on the basis of the described effect of facial masculinity or risk aversion [10]. However, our results show that other mechanisms are likely to be at work. This conjecture is consistent with the lack of correlation that we observe between bidding behaviour and Index 3 [49]. This measure of masculinity has been described to be related with risk taking in an investment game [10]. Behaviour displayed in both experiments, investment and bidding, are related to risk attitudes [10]; [60]–[62], [90]–[92], [94]. But there is a crucial difference between the two. The auction is a strategic game where players must consider the decision of others. On the other hand, individual payoffs in the investment game depend only on participants' own decisions and on chance [10].

At this point, it is important to notice that bidding behaviour displays a weakly significant correlation with the standard measure of risk aversion proposed by Holt and Laury [85], which uses a set of 10 pairs of lottery choices (a non-strategic situation like the investment game [10]). In addition, the correlations found between bidding behaviour and facial measures of masculinity were weak. However, when we control for risk aversion, the relationships between bid and facial measures of masculinity become strong. All this indirect evidence suggests that variables other than risk aversion could be influencing bidding behavior in our first-price auction. As pointed out by other authors under different auction formats [68]–[71], bidding behaviour seems to be related to competitiveness. Thus, competitiveness might be also driving our results, even though social context is of relative little relevance in our auction format. Clearly, the influence of masculinity on bidding behaviour through competitiveness deserves further study in standard private value auctions (where valuations are independent and participants can only bid once). It would have also been interesting to measure risk aversion with an investing game in order to obtain a broader picture of the possible relation between the determinants of bidding behaviour and masculinity.

Regarding facial trustworthiness, it correlates negatively with fWHR and EME, and positively with Nw and Index 3. The negative correlation with fWHR is in line with the results obtained by Stirrat and Perrett [32]: more masculine faces are less trustworthy. This result however contrasts with the negative correlation between trustworthiness scores and EME, which we expected to be positive, and with the positive correlation between trustworthiness scores and Nw and Index 3, since higher values of these measures are associated with more masculine faces. Thus, masculinity measured with EME, Nw and Index 3 is positively correlated with trustworthiness. This positive correlation has been observed by other authors under different conditions: Macapagal and collaborators [95] found that hypermasculinity scores obtained through a questionnaire were correlated to trustworthiness scores given by other males, whereas Thompson and O'Sullivan [96] found no correlation between facial masculinity and trustworthiness when women were rating males' trustworthiness. We thus find conflicting results on the relationship between masculinity and trustworthiness, in line with previous studies. Nevertheless, it is important to note that the sexual dimorphism of fWHR is currently being questioned [55]–[58]. This cast doubts on the idea that the positive relation between trustworthiness and fWHR found in the literature is reflecting a link between testosterone exposure during adolescence and trustworthiness. In other words, fWHR might not be measuring masculinity because a masculine physical trait should be necessarily different between males and females. In any case, one possible explanation for all these conflicting results might be that the perception of trustworthiness is actually mediated by a third (uncontrolled) variable in different ways across populations. For example, it has been shown that perceived aggressiveness interferes with the relationship between masculinity and attractiveness [16].

One interesting result is that many measures of masculinity do not correlate with the perceived masculinity score. This is remarkable as both measured and perceived masculinity affect human behaviour [10], [40], [42], [43], [50]. As a matter of fact, they are often used as synonymous concepts. This puzzling result has been observed before [15], [50]. Several reasons may explain why these measures are at odds, but it is quite plausible that subjective judgments of masculinity are influenced by other factors apart from just the morphology of the face. For example, it is important to notice that perceived masculinity is a positive predictor of male attractiveness whereas measured masculinity is not [39], [97]. Raters perceive attractive images as masculine, maybe due to stereotypical associations between both characteristics [50] or because of the correlation between perceived masculinity and other desirable features such as perceived health [39]. In short, attractiveness might influence perceived masculinity. Another possibility is that raters do not perceive masculinity as a measure of the differences between sexes, a condition more or less explicitly shared by all masculinity measures, but rather as a measure of differences among males. If that were the case, raters might perceive facial masculinity as a feature that correlates with other features considered as masculine such as dominance or interest in sex [22]–[27]. This explanation is plausible given that some of these behaviours are linked to the current T level, that in turn shows a correlation with perceived masculinity [41], [42]. The analysis of the relationship between facial masculinity measures and behaviours considered to be masculine deserves further exploration that will help to clarify the relationship between measured and estimated masculinity. In any case, the present study shows that variables reflecting differences in facial shape between males and females, possibly linked to hormonal differences during development, do have an impact on behaviour.

At this point it is important to notice that not all the measures of masculinity that we considered here suffer from a lack of correlation with perceived masculinity. In fact, our results confirm previously described correlations between perceived and morphometric measures of masculinity. Perceived masculinity correlates with Index 1, Index 2 (contrary to what was found in [50]), Index 3, DiscSco1 (contrary to what was found in [39]), DiscSco2 (as previously found in [52]), and LLh [33]. Surprisingly, not all the variables that show values significantly different between males and females are related to perceived facial masculinity. Men with “feminine” values of fWHR, EME, ULh, Nw and ProcDist are not perceived as less masculine by others. Furthermore, LLh does not show sexual dimorphism in our sample but shows a positive correlation with perceived masculinity. The reason behind these differences in the association between sexually dimorphic variables and perceived masculinity may be that raters focus on just a few features when classifying a face as masculine or not. If a particular method of measuring masculinity includes any of these features, a correlation will arise. Hence, our results do not run against the properties commonly exhibited by morphological and perceived measures of masculinity [38], given that perceived facial masculinity surely depends on some features that differ from males to females. This tendency to focus on a single feature is independent of whether raters understand masculinity as a difference between males and females or as a facial feature related to other masculine traits.

We take into account that the total number of statistical analyses employed in this work could be affecting the results, since it increases the probability of obtaining type I and II errors. Let us reiterate that the aim of our analyses was not to reproduce individually the experiments previously carried out by other authors, nor to uncover new effects, but to make an informed comparison of a wide variety of measurement methods. We have performed an exhaustive comparative analysis between methods, although we are aware of the relatively low statistical power of our results. Therefore, we must stress the fact that the novelty and usefulness of our work does not reside in the statistically significant results, but in the comparison of the relationship between behavior and the measures of facial masculinity considered.

Finally, it would be useful for the development of this field to adopt a policy of full data availability, which ourselves are willing to adopt (conditional on ethical constraints). The use of as many measurement methods as possible on subjects from previous experiments would increase total sample size. This would also allow researchers to contrast their results with those obtained when analysing different behaviours in different setups. Such policy would help to standardise the definition and measurement of facial masculinity, and ultimately clarify its influence on behaviour.

## Conclusions

The main aim of our work was to compare several measures of facial masculinity by studying their relation with perceived masculinity, bidding behaviour and trustworthiness. Results previously obtained in the literature are often unclear or contradictory and they do not help to clarify the influence of facial masculinity on behaviour. Researchers in the field do not currently have a solid starting point. We have shown that perceived masculinity correlates with many, but not all, the measurements we considered. Bidding behaviour did not correlate with most measures, only with those employing Geometric Morphometrics. This suggests that methods which consider the whole facial shape might be well suited to study the relationship between masculinity and strategic behaviour. Since there could be variables influencing bidding behaviour other than risk aversion, we controlled for the effect of Holt and Laury's measure of risk aversion. We found the relationship between masculinity and bidding behavior to become stronger, an interesting result that should be further studied. Finally, we only found the expected correlation between masculinity and trustworthiness when considering fWHR, which on the other hand has been shown not to be a sexually dimorphic feature (and thus, not a proper masculinity measure). The rest of masculinity measures showed the opposite correlation to the one expected or no correlation at all. All this suggests that factors other than exposure to T during adolescence could be interfering in the perception of trustworthiness. We suggest that it could be useful to apply all these measurements methods to previous studies carried out by different authors in order to compare results and increase sample size.

## Supporting Information

Appendix S1 **Additional analyses regarding the robustness of sample-dependent measures, the correlation among masculinity measures and the correlation between Holt and Laury measure of risk aversion and masculinity measures.**
(DOCX)

Dataset S1 **Behaviour and masculinity scores obtained for each of the 147 participants.**
(XLSX)
