# Supplementary material for: Facial Masculinity: How the Choice of Measurement Method Enables to Detect Its Influence on Behaviour
Source: PLoS One. 2014 Nov 12;9(11):e112157. doi: 10.1371/journal.pone.0112157 (PMC4229127; doi:10.1371/journal.pone.0112157)
Supplement: Appendix S1 — Additional analyses regarding the robustness of sample-dependent measures, the correlation among masculinity measures and the correlation between Holt and Laury measure of risk aversion and masculinity measures. (DOCX) [file pone.0112157.s001.docx]

**APPENDIX S1**

**1- ROBUSTNESS OF SAMPLE-DEPENDENT MEASURES**

Three of the measures we employ in this study are sample-dependent (ProcDist, DiscSco1 and DiscSco2). We checked the robustness of these measures to changes in their method of calculation, i.e. to what extent sample-dependent measures of masculinity vary with changes in the exact method of calculation. To this end, we changed both the female face of reference and the set of landmarks employed to define the facial shape.

***Changes in the female face of reference***

Firstly we checked the robustness of ProcDist, DiscSco1 and DiscSco2 by changing the reference female face. We generated three new reference female faces (faces A, B and C). These faces were constructed by averaging subsamples of our initial female sample (n=74). The photos in each subsample were chosen randomly. A and B subsamples included 25 photos and the C pool included 24.

The steps followed in order to calculate DiscSco1 with each of the three reduced samples of females separately are shown in Table S1. To calculate these measures, MorphoJ superimposed, for each of the subsamples, the shapes with a generalized least-squares Procrustes fit. The covariance matrix was computed from these data of variation among individuals, and a PCA was carried out on it. Then, for subsequent analyses, we chose all the PCs with eigenvalues higher than the average. Step-wise discriminant analysis was then used to choose among the PCs which of them were best able to discriminate between sexes. These discriminant function scores are used as an index of masculinity and compared with the initial computed DiscSco1 and with the bidding behaviour.

For DiscSco2, the three masculinity values was calculated employing the same ten different facial measures (Chin length, Eye height, Eye width, Interpupil distance, Lip height, Lip width, Jaw width, Face width, Face length and Face length minus chin). The steps followed for calculating DiscSco2 with each of the three subsamples are shown in Table S1. First, we computed the unstandardized residuals of the first eight of these facial measures on the correspondent face lengths (the last three facial measures) to control for face size. We examined sex differences in these eight residual variables by GLM (controlling for age) for each subsample. Then we performed a principal axis factor analysis on the significant different variables. We chose the major factors (which accounted for, at least, 70% of variation). The considered major factors were rotated (varimax) and extracted for each subsample. The factors which significantly discriminated between sexes were entered into a discriminant analysis predicting sex. These discriminant function scores were compared with the DiscSco2 score calculated initially as measure of facial masculinity and also with the bidding behavior.

To calculate ProcDist we simply calculated the Procrustes distance between the shape of the symmetrized participant`s faces (males) and three new female reference faces. These three distances are also compared with the initial ProcDict score and with the bidding behavior.

The three alternative reference faces generate ProcDist scores which correlate strongly with those obtained by using the initial reference face (Table S2). The correlation coefficients of the scores computed with the reduced samples with the original DiscSco1 and DiscSco2 measures are slightly lower than the one obtained for ProcDist (Table S2). Table S2 also shows the correlations of the three alternative measures of ProcDist, DiscSco1 and DiscSco2 with bid. All ProcDist alternative measures maintain a significant correlation, while one of the alternative DiscSco1 calculations loses its correlation with the bids.

The results suggest that sample-dependent measures of masculinity are relatively independent of the sample employed to build the female reference face. It seems that 20 faces are enough to build a correct reference female face. It is important to note that ProcDist is the measure which is less sensitive to the sample. DiscSco1 and DiscSco2, although also weakly affected by changes in the female reference sample, show lower correlations with their respective original measure (the one constructed with the whole female sample). This suggests that ProcDist is more robust to changes in the female sample of reference.

***Changes in the number of LMs***

Secondly, we checked the robustness of ProcDist by varying the number of LMs employed to define the shapes. We sequentially excluded the LMs which showed larger deviations (see Figure 1). More specifically, we excluded the LMs in the insertion of the neck (LMs #38 and #39), the eyebrows (LMs from #30 to #37), the jaw (LMs #28 and #29) and the chin (LM #27). Therefore, we successively defined the facial shape with 37, 29, 27 and 26 LMs respectively. A discriminant function employing these four LMs configurations correctly classified the gender of the faces in the sample in the following percentages: 94.12% for the 37LMs shape (T^2^=816.7997; p<0.0001), 89.59% for the 29LMs shape (T^2^=447.2662; p<0.0001), 89.14% for the 27LMs shape (T^2^=408.5013; p<0.0001) and 86.88% for the 26LMs shape (T^2^=1385.2776; p<0.0001).

The Procrustes distances computed by using a reduced number of LMs produced scores which also strongly correlate with the original measure that included all 39LMs (Table S3). However, the correlation between the masculinity scores and the bid weakens progressively as the number of LMs employed goes down. Only the correlation between the 37LMs score and the bid remains close to statistical significance (Table S3).

Thus, as it is shown here, the progressive reduction in the number of LMs decreases the power of ProcDist to explain bidding behavior. One possible reason for this is that by eliminating some of the LMs we also eliminated features that relate to face width (LM#28-29 or even LM#38-39) and length (LM#29 and LMs of the eyebrow). These two features, face length and width, are very important when defining masculinity (Weston et al. 2007; Carré and McCormick 2008; Stirrat and Perrett 2010). They are included in most of the methods of measuring masculinity that employ traditional morphometrics (i.e., EME angle depends on these two measures). The shape defined exclusively by eyes, lips and nose, but which excludes these two features, does not correlate with bidding behavior, even though gender differences persist when shapes are defined using fewer LMs. Therefore, a small change in the number of LMs does not seem to affect this measure much, although removing a few specific LMs has a substantial impact on its predictive value. Future studies should condition their LMs selection not only on their morphological properties, but also on the variables under study. When choosing LMs, morphologists strongly recommend that 1) LMs have to be located precisely on each specimen under study and 2) that LMs have to show a clear one-to-one correspondence from specimen to specimen. This correspondence clearly depends on the context of study and does not necessarily imply strict homology across LMs (Klingenberg 2008; Polly 2008).

**Table S1.** Procedures followed to calculate DiscSco1 and DiscSco2 when we employed a reduced sample of females instead the whole population.

|  |  | **SUBSAMPLE A** | **SUBSAMPLE B** | **SUBSAMPLE C** |
| --- | --- | --- | --- | --- |
| **DiscSco1** | **Number of PCs choose and % of variance accounted by them** | The first eight PCs (which together accounted for 89.33%) | The first ten PCs (which together accounted for 87.31%) | The first eleven PCs which together accounted for 89.26% of the variance in facial landmark configuration |
|  | **Number of PCs incorporated in the discriminant function and % of correct classification.** | The resulting discriminant function incorporated five of the PCs and correctly clasificate 97.10% of faces (low values correspond to males) | The resulting discriminant function incorporated four of the PCs and correctly clasificate 94.2% of faces (low values correspond to males). | The resulting discriminant function incorporated four of the PCs and correctly clasificate 95.9% of faces. In this case, low values correspond to females. |
| **DiscSco2** | **Which of the eight residual variables are significant different between sexes?** | interpupil distance, face width, chin length, jaw width, eyes length and eyes width | interpupil distance, chin length, jaw width, eyes height and lips width | interpupil distance, face width, chin length, jaw width, eyes length and eyes width |
|  | **Factors which accounted for at least 70% of variation (and their eigenvalues)** | Three major factors accounted for 75.98% of variation. Their eigenvalues was 1.981, 1.526 and 1.015. | Three major factors accounted for 78.13% of variation. The eigenvalues was 1.878, 1.218 and 0.811. | Three major factors accounted for 83.74% of variation. Their eigenvalues was 1.847, 1.419 and 0.920. |
|  | **Contribution of the variables on the major factors** | PC1: primarily defined by eye height (0.770), eye width (0.675), chin length (-0.641) and, in a lesser stent, interpupil distance (0.566).  PC2: face width (-0.755), jaw width (0.532) and interpupil distance (0.529).  PC3: jaw width (0.520) and face width (0.519) | PC1: chin length (-0.813) and jaw width (-0.672)  PC2: lip width (0.664) and interpupil distance (0.661)  PC3: eye height (0.519) | PC1: eye height (0.791), chin length (-0. 759) and jaw width (-0.600)  PC2: face width (-0.769) and eye width (0.760)  PC3: face width (0.572) |
|  | **Does the major factors discriminate between sexes?** | PC1: F_1,170_=15.635, **p<0.001**  PC2: F_1,170_=7.744, **p=0.006**  PC3: F_1,170_=1.845, p=0.176 | PC1: F_1,170_=12.655, **p<0.001**  PC2: F_1,170_=7.557, **p=0.006**  PC3: F_1,170_=1.953, p=0.164 | PC1: F_1,169_=8.363, **p=0.004**  PC2: F_1,169_=6.971, **p=0.009**  PC3: F_1,169_=3.205, p=0.075 |
|  | **Percentage of faces correctly classified by the Discriminant function** | 87.8%  (low discriminant function scores correspond to males) | 88.4%  (low discriminant function scores correspond to females) | 87.7%  (low discriminant function scores correspond to males) |

**Table S2.** Correlations between the three sample-dependent measures of masculinity after changes in the female face of reference and the original measure (i.e. using the whole female sample) and bidding behavior.

|  | **ProcDist** | | **DiscSco1** | | **DiscSco2** | |
| --- | --- | --- | --- | --- | --- | --- |
|  | **Masculinity Whole sample** | **Bid** | **Masculinity Whole sample** | **Bid** | **Masculinity Whole sample** | **Bid** |
| **Masculinity Subsample A** | r=0.985 | σ =-0.166 | r=0.962 | σ =0.173 | r=0.903 | σ =0.081 |
|  | p<0.001 | p=0.044 | p<0.001 | p=0.037 | p<0.001 | p=0.328 |
| **Masculinity Subsample B** | r=0.992 | σ =-0.165 | r=0.961 | σ =0.137 | r=-0.654 | σ =0.035 |
|  | p<0.001 | p=0.046 | p<0.001 | p=0.099 | p<0.001 | p=0.672 |
| **Masculinity Subsample C** | r=0.996 | σ =-0.172 | r=-0.977 | σ =-0.170 | r=0.967 | σ =0.076 |
|  | p<0.001 | p=0.038 | p<0.001 | p=0.040 | p<0.001 | p=0.359 |

**Table S3.** Correlations between the ProcDist measure of masculinity after reducing the number of landmarks employed and the original measure (i.e. using the whole female sample) and bidding behavior.

|  | **Masculinity Whole sample** | **Bid** |
| --- | --- | --- |
| **ProcDist**  **Shape defined by 37 LMs** | r=0.760 | σ =-0.161 |
|  | p<0.001 | p=0.052 |
| **ProcDist**  **Shape defined by 29 LMs** | r=0.682 | σ =-0.090 |
|  | p<0.001 | p=0.276 |
| **ProcDist**  **Shape defined by 27 LMs** | r=0.432 | σ =-0.068 |
|  | p<0.001 | p=0.414 |
| **ProcDist**  **Shape defined by 26 LMs** | r=0.393 | σ =-0.040 |
|  | p<0.001 | p=0.635 |

**2- CORRELATION AMONG MASCULINITY MEASURES**

Our results suggest that different masculinity measures do not measure the same features. Thus, we believe that it would be very useful to report the correlation between the different masculinity measures, as shown in Table S4. This might give some pointers to the similarities and differences among these measures. Still, a deeper morphometric analysis would be required in order to clarify why some masculinity measures do not correlate with each other.

**Table S4.** Coefficients of correlation among all the morphometric measures of masculinity described in the study.

|  | **fWHR** | **Ln ULh** | **LLh** | **Nw** | **EME** | **Index 1** | **Index 2** | **Index 3** | **ProcDist** | **DiscSco1** | **DiscSco2** |
| --- | --- | --- | --- | --- | --- | --- | --- | --- | --- | --- | --- |
| **fWHR** | 1 | r=0.079 | r=-0.025 | r=-0.057 | r=0.750 | r=-0.291 | r=-0.261 | r=-0.188 | r=0.228 | r=-0.275 | r=-0.196 |
|  |  | p=0.344 | p=0.765 | p=0.492 | **p<0.001** | **p<0.001** | **p=0.001** | **p=0.023** | **p=0.005** | **p=0.001** | **p=0.018** |
|  | **Ln ULh** | 1 | r=0.435 | r=0.166 | r=-0.180 | r=-0.006 | r=0.205 | r=0.352 | r=0.022 | r=-0.087 | r=-0.069 |
|  |  |  | **p<0.001** | **p=0.045** | **p=0.030** | p=0.964 | **p=0.013** | **p<0.001** | p=0.791 | p=0.294 | p=0.408 |
|  |  | **LLh** | 1 | r=0.117 | r=-0.171 | r=0.180 | r=-0.050 | r=-0.064 | r=-0.054 | r=0.055 | r=0.320 |
|  |  |  |  | p=0.158 | **p=0.038** | **p=0.029** | p=0.546 | p=0.442 | p=0.519 | p=0.510 | **p<0.001** |
|  |  |  | **Nw** | 1 | r=-0.273 | r=0.054 | r=0.036 | r=0.131 | r=0.001 | r=-0.090 | r=-0.004 |
|  |  |  |  |  | **p=0.001** | p=0.584 | p=0.666 | p=0.112 | p=0.990 | p=0.277 | p=0.963 |
|  |  |  |  | **EME** | 1 | r=-0.347 | r=-0.057 | r=-0.179 | r=0.129 | r=-0.137 | r=0.001 |
|  |  |  |  |  |  | **p<0.001** | p=0.495 | **p=0.030** | p=0.119 | p=0.098 | p=0.991 |
|  |  |  |  |  | **Index 1** | 1 | r=0.091 | r=-0.011 | r=-0.004 | r=0.068 | r=0.306 |
|  |  |  |  |  |  |  | p=0.273 | p=0.841 | p=0.959 | p=0.410 | **p<0.001** |
|  |  |  |  |  |  | **Index 2** | 1 | r=0.722 | r=-0.052 | r=-0.233 | r=-0.261 |
|  |  |  |  |  |  |  |  | **p<0.001** | p=0.528 | **p=0.004** | **p=0.001** |
|  |  |  |  |  |  |  | **Index 3** | 1 | r=-0.045 | r=-0.228 | r=-0.473 |
|  |  |  |  |  |  |  |  |  | p=0.591 | **p<0.001** | **p<0.001** |
|  |  |  |  |  |  |  |  | **ProcDist** | 1 | r=-0.617 | r=-0.106 |
|  |  |  |  |  |  |  |  |  |  | **p<0.001** | p=0.200 |
|  |  |  |  |  |  |  |  |  | **DiscSco1** | 1 | r=0.256 |
|  |  |  |  |  |  |  |  |  |  |  | **p=0.002** |
|  |  |  |  |  |  |  |  |  |  | **DiscSco2** | 1 |
|  |  |  |  |  |  |  |  |  |  |  |  |

**3- HOLT AND LAURY’S RISK AVERSION AND MEASURES OF MASCULINITY**

For completeness, we present the correlation between the Holt and Laury’s measure of risk aversion and all the measures of facial masculinity considered in our study (Table S5).

**Table S5.** Correlations between perceived masculinity and morphometric masculinity measures with Holt & Laury risk aversion (n=141).

|  | **Risk Aversion** |
| --- | --- |
| **Age** | **ρ=-0.232** |
|  | **p=0.006** |
| **fWHR** | ρ=0.085 |
|  | p=0.317 |
| **EME** | ρ=-0.028 |
|  | p=0.745 |
| **Ln ULh** | ρ=0.002 |
|  | p=0.985 |
| **LLh** | ρ=0.036 |
|  | p=0.673 |
| **Nw** | ρ=0.051 |
|  | p=0.552 |
| **Index 1** | ρ=0.056 |
|  | p=0.510 |
| **Index 2** | ρ=-0.061 |
|  | p=0.469 |
| **Index 3** | ρ=-0.050 |
|  | p=0.555 |
| **ProcDist** | ρ=0.057 |
|  | p=0.503 |
| **DiscSco1** | ρ=-0.044 |
|  | p=0.602 |
| **DiscSco2** | ρ=-0.044 |
|  | p=0.602 |
| **Perceived Masculinity** | ρ=-0.128 |
|  | p=0.130 |

**REFERENCES**

Carré JM, McCormick CM (2008) In your face: Facial metrics predict aggressive behaviour in the laboratory and in varsity and professional hockey players. Proceedings of the Royal Society B: Biological Sciences 275: 2651–2656.

Klingenberg CP (2008) Novelty and ‘‘Homology-free’’ Morphometrics: What’s in a Name? Evolutionary Biology 5: 186–190.

Polly PD (2008) Developmental dynamics and G-matrices: Can morphometric spaces be used to model phenotypic evolution? Evolutionary Biology 35: 83–96.

Stirrat M, Perrett DI (2010) Valid facial cues to cooperation and trust: male facial width and trustworthiness. Psychological Science 21: 349–354.

Weston EM, Friday AE, Lio P (2007) Biometric evidence that sexual selection has shaped the hominin face. PLoS ONE 2: e710.
